# Atypical Lemierre's Syndrome Presenting With Extensive Neurovascular Complications and Multifocal Ischemic Stroke

**DOI:** 10.7759/cureus.117555

**Published:** 2026-10-06

**Authors:** Hayoung E Ahn, Juan P Villablanca, Jonathan F Garcia

**Affiliations:** 1 Internal Medicine, UCLA David Geffen School of Medicine, Los Angeles, USA; 2 Radiology, UCLA Health, Los Angeles, USA; 3 Internal Medicine, UCLA Health, Los Angeles, USA

**Keywords:** cerebral venous sinus thrombosis, clival osteomyelitis, fusobacterium necrophorum, ischemic stroke, lemierre’s syndrome

## Abstract

Lemierre’s syndrome is a rare condition classically characterized by septic thrombophlebitis of the internal jugular vein following an oropharyngeal infection. We report a case of a previously healthy 19-year-old man who initially presented with gastrointestinal and constitutional symptoms (nausea, vomiting, diarrhea, fever, and malaise) and thrombocytopenia and was treated with corticosteroids for presumed immune thrombocytopenia, with subsequent neurological deterioration associated with carotid artery occlusion, venous sinus thrombosis, and multifocal cerebral infarctions. Further evaluation revealed skull base osteomyelitis, meningitis, and septic pulmonary emboli. Microbial cell-free DNA (mcfDNA) testing identified *Fusobacterium necrophorum*, leading to a diagnosis of Lemierre’s syndrome. The patient underwent surgical debridement and received prolonged intravenous antibiotics and anticoagulation, with gradual clinical improvement. This case highlights an atypical presentation of Lemierre’s syndrome lacking the classic pharyngeal symptoms and emphasizes the importance of early recognition in patients with unexplained thrombosis and neurological complications.

## Introduction

Lemierre’s syndrome is a rare but severe infection most commonly caused by *Fusobacterium necrophorum*, characterized by septic thrombophlebitis of the internal jugular vein and metastatic septic emboli [1]. Although classically associated with antecedent oropharyngeal infection, presentations may be variable and involve multiple organ systems due to hematogenous dissemination and contiguous spread. Neurological complications, including cerebral venous sinus thrombosis, arterial occlusion, and ischemic stroke, are uncommon but associated with significant morbidity [2]. Diagnostic delays may occur when initial symptoms are nonspecific or mimic other conditions. This case illustrates the uncommon combination of extensive arterial and venous neurovascular involvement, skull-base infection, and multifocal ischemic stroke in a young patient without preceding pharyngeal symptoms. The initial gastrointestinal symptoms and thrombocytopenia obscured the infectious diagnosis, highlighting the need to reassess an initially noninfectious explanation when neurological deficits and thrombosis emerge.

## Case presentation

A previously healthy 19-year-old man presented with several days of gastrointestinal symptoms, including nausea, vomiting, and diarrhea, associated with fever and progressive malaise following recent travel. He was admitted to a community hospital, where initial laboratory testing demonstrated severe thrombocytopenia, leukocytosis, and acute kidney injury (Table 1). He was treated with corticosteroids for presumed immune thrombocytopenia. At this stage, the gastrointestinal symptoms, fever, thrombocytopenia, leukocytosis, and acute kidney injury were nonspecific and did not establish a unifying diagnosis.

**Table 1:** Selected laboratory findings by encounter and specimen The community-hospital emergency department visits occurred eight days apart; the tertiary-center lumbar puncture occurred later. CSF cell count and differential represent one of two samples from the same lumbar puncture; glucose and protein were reported for tube 1. Reference intervals are representative adult intervals, not source-laboratory intervals, and may vary by laboratory. ED, emergency department; CSF, cerebrospinal fluid; H, above the listed reference range; L, below the listed reference range

Encounter and specimen	Test	Result	Reference range
Initial community ED visit - blood	White blood cell count (×10⁹/L)	24.8 (H)	4.5-11.0
Initial community ED visit - blood	Platelet count (×10⁹/L)	40 (L)	150-400
Initial community ED visit - blood	Creatinine (mg/dL)	5.2 (H)	0.60-1.30
Initial community ED visit - blood	Blood urea nitrogen (mg/dL)	59 (H)	7-22
Return community ED visit - blood	White blood cell count (×10⁹/L)	11.3 (H)	4.5-11.0
Return community ED visit - blood	Platelet count (×10⁹/L)	130 (L)	150-400
Return community ED visit - blood	Creatinine (mg/dL)	0.7	0.60-1.30
Return community ED visit - blood	Blood urea nitrogen (mg/dL)	20	7-22
Tertiary-center lumbar puncture - CSF	Nucleated cell count (cells/µL)	45 (H)	0-5
Tertiary-center lumbar puncture - CSF	Lymphocytes (%)	94	Mononuclear cells normally predominate
Tertiary-center lumbar puncture - CSF	Neutrophils (%)	1	0 expected in normal adult CSF
Tertiary-center lumbar puncture - CSF	Monocytes (%)	5	Mononuclear cells normally predominate
Tertiary-center lumbar puncture - CSF	Glucose (mg/dL)	39 (L)	50-80
Tertiary-center lumbar puncture - CSF	Protein (mg/dL)	135 (H)	15-60

Approximately three days after discharge from the initial hospitalization, the patient re-presented with persistent headache and acute left upper limb weakness. Neuroimaging demonstrated occlusion of the left internal carotid artery (Figures 1, 2), abnormalities of the right internal carotid artery, and bilateral watershed infarctions (Figure 3). His course was further complicated by fever and identification of a retropharyngeal fluid collection. An initial lumbar puncture at an outside hospital was reported to show pleocytosis with a mixed lymphocytic-neutrophilic profile; Gram stain, culture, and cryptococcal antigen were reported as negative. Chest computed tomography at the outside hospital revealed bilateral pulmonary nodules. A CT-guided lung biopsy during that admission showed acute inflammation and necroinflammatory debris without evidence of vasculitis; fungal and acid-fast bacilli stains were negative. The patient subsequently developed progressive thrombotic complications involving the cerebral venous system and was transferred to a tertiary care center for further management.

**Figure 1:**
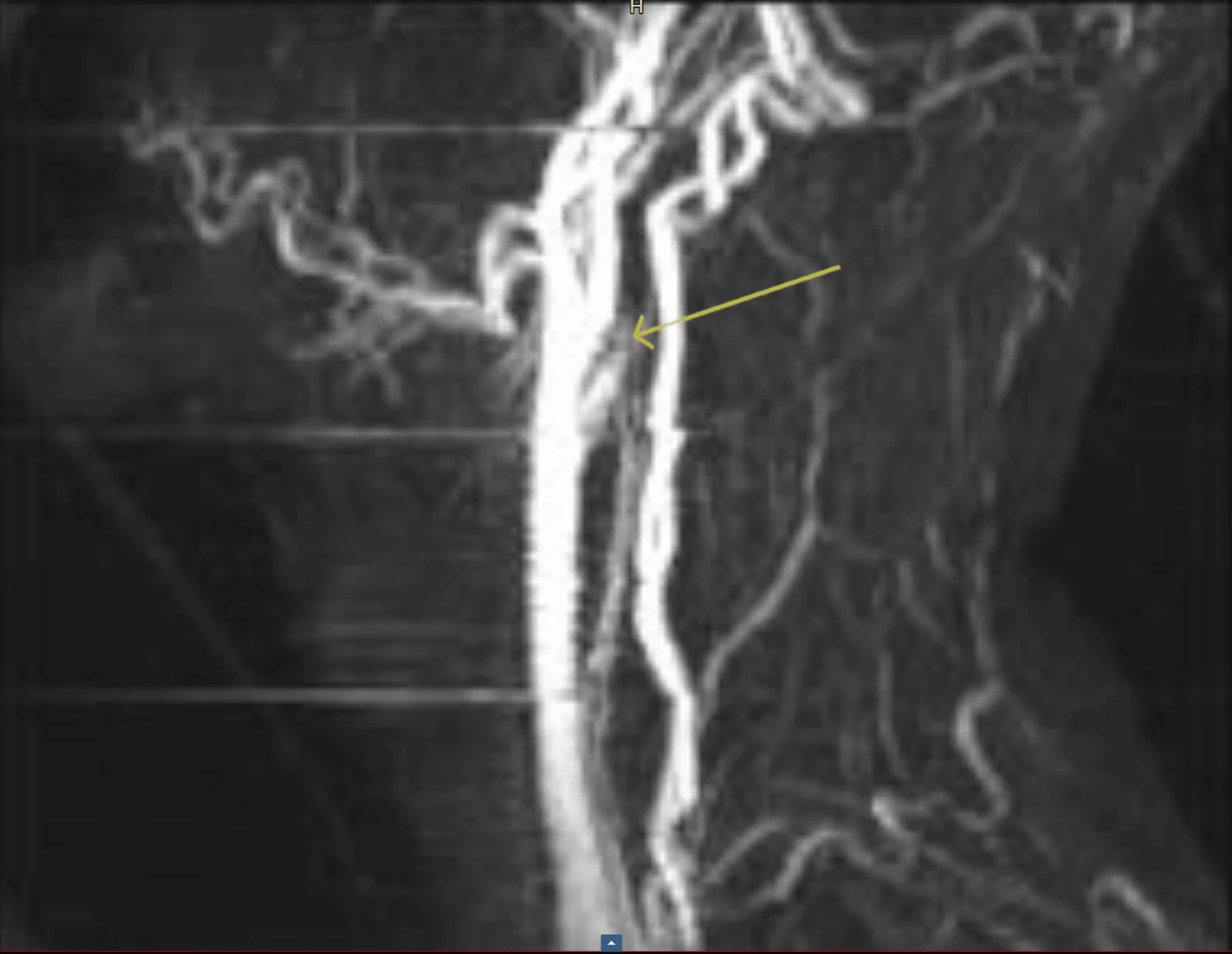
Sagittal MRA, with the arrow indicating near-occlusive stenosis of the left ICA. The stenosis transitions to occlusion in the high cervical and intracranial ICA, with reconstitution of flow in the terminal ICA MRA, magnetic resonance angiography; ICA, internal carotid artery

**Figure 2:**
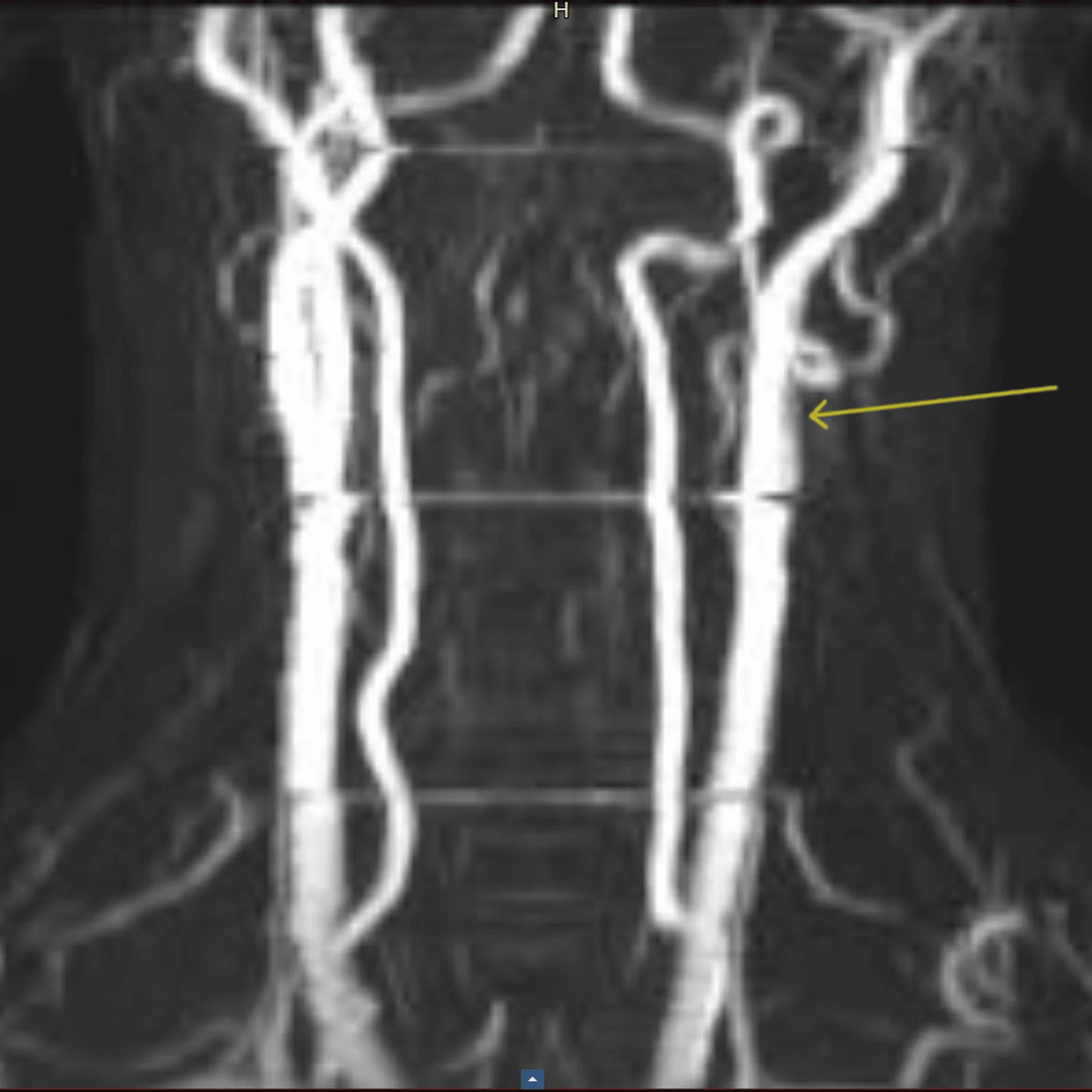
Coronal MRA, with the arrow indicating near-occlusive stenosis of the left ICA. The stenosis transitions to occlusion in the high cervical and intracranial ICA, with reconstitution of flow in the terminal ICA MRA, magnetic resonance angiography; ICA, internal carotid artery

**Figure 3:**
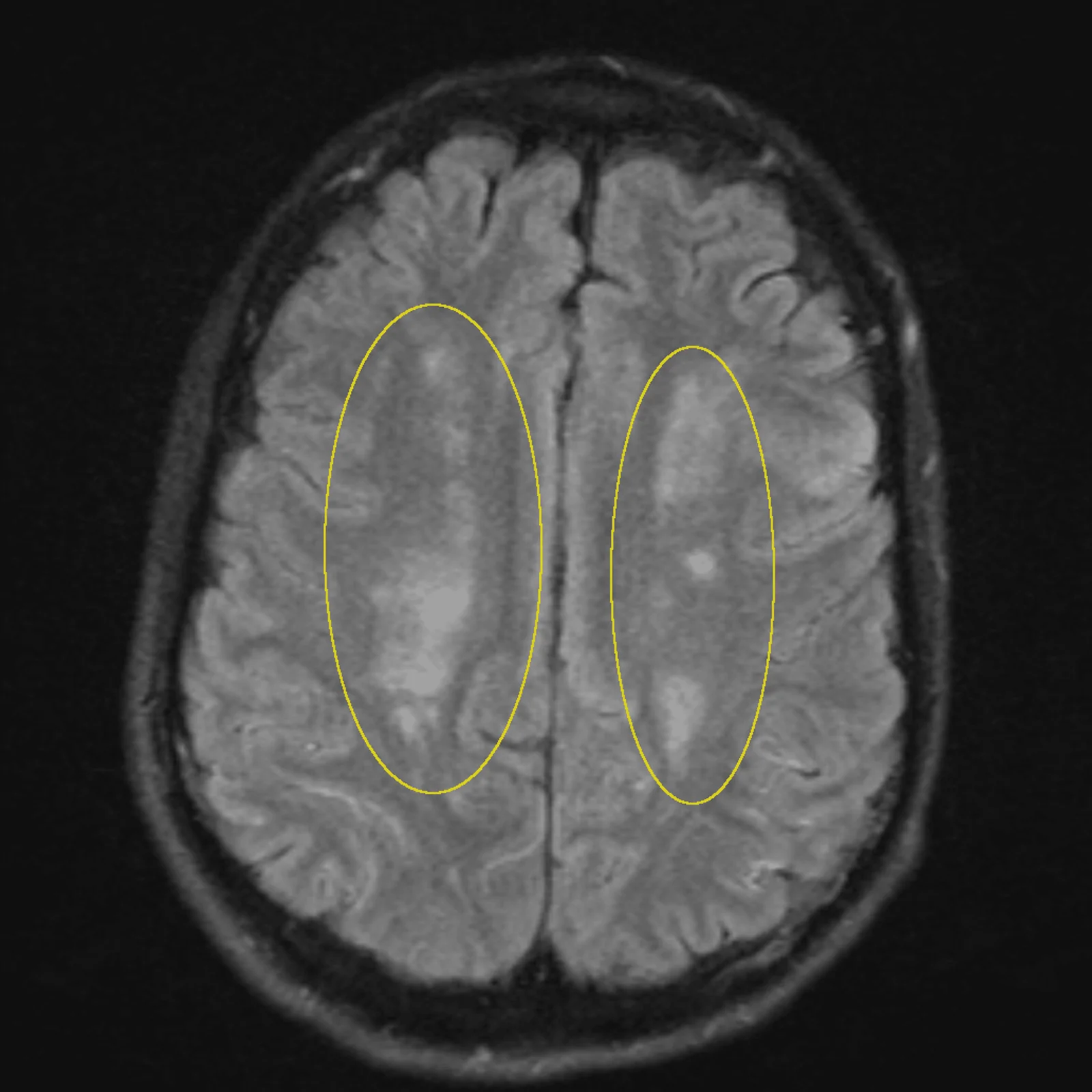
Axial FLAIR MRI, with the oval outlines highlighting bilateral watershed (borderzone) infarcts FLAIR MRI, fluid-attenuated inversion recovery magnetic resonance imaging

After transfer to the tertiary care center, initial investigations were suggestive of a disseminated infectious or inflammatory process with central nervous system involvement. Repeat cerebrospinal fluid analysis showed pleocytosis (45 nucleated cells/µL; 94% lymphocytes, 1% neutrophils, and 5% monocytes), decreased glucose, and increased protein, consistent with meningitis (Table 1).

Magnetic resonance imaging of the brain and spine demonstrated clival osteomyelitis with an associated epidural abscess, as well as infectious vasculopathy involving the internal carotid arteries and cerebral venous system, including cavernous sinus thrombosis. Computed tomography angiography of the head and neck demonstrated skull base osteomyelitis involving the basisphenoid and basiocciput, a prepontine epidural abscess, inflammatory narrowing of the vertebrobasilar arteries, and thrombosis of the right sigmoid sinus, jugular bulb, and internal jugular vein (Figures 4, 5). Digital subtraction angiography confirmed left internal carotid artery occlusion, multifocal narrowing of the right internal carotid and basilar arteries, and thrombosis of the internal jugular and sigmoid sinuses (Figure 6).

**Figure 4:**
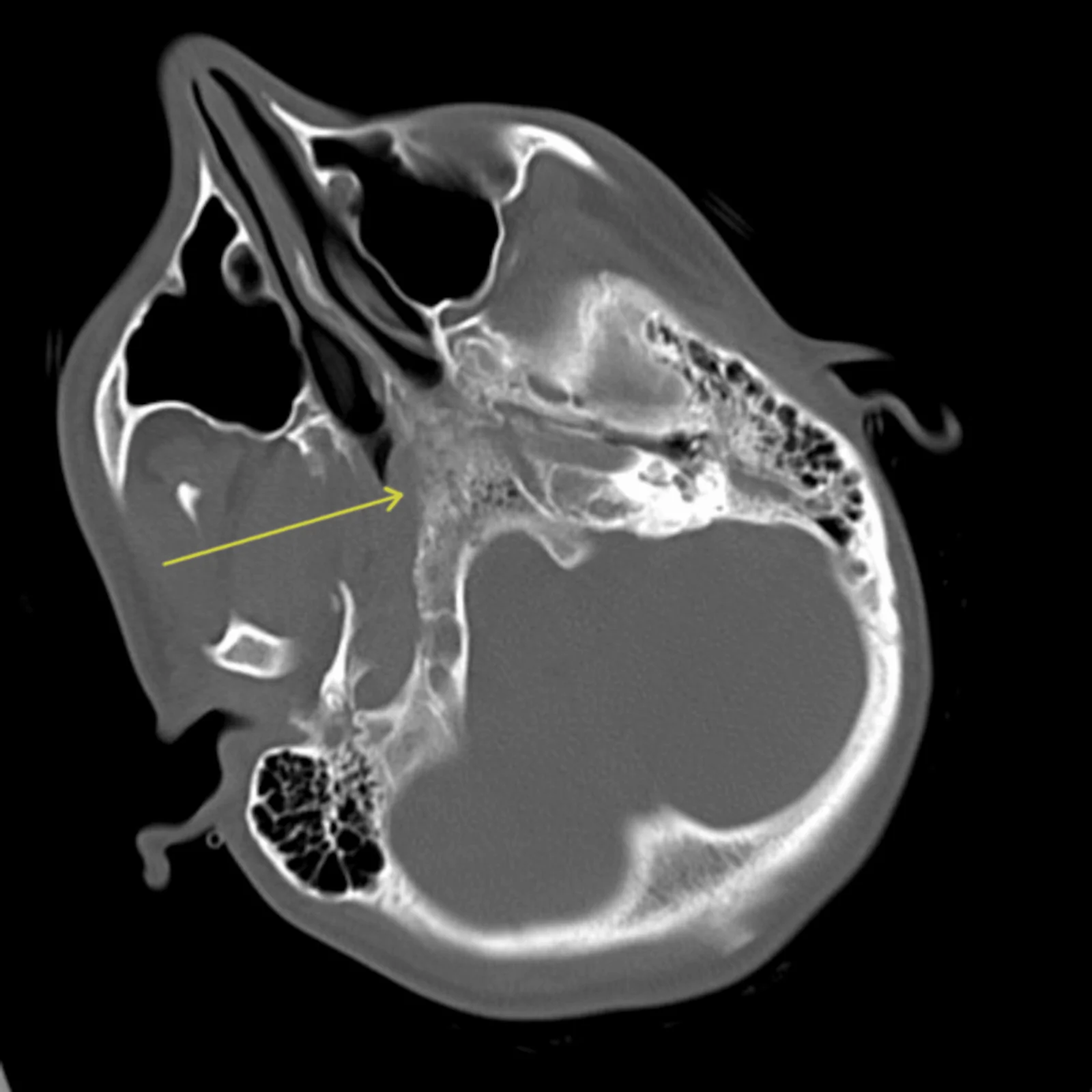
Axial CT, with the arrow indicating extensive central bony destruction of the clivus with pockets of intraosseous gas, consistent with osteomyelitis CT, computed tomography

**Figure 5:**
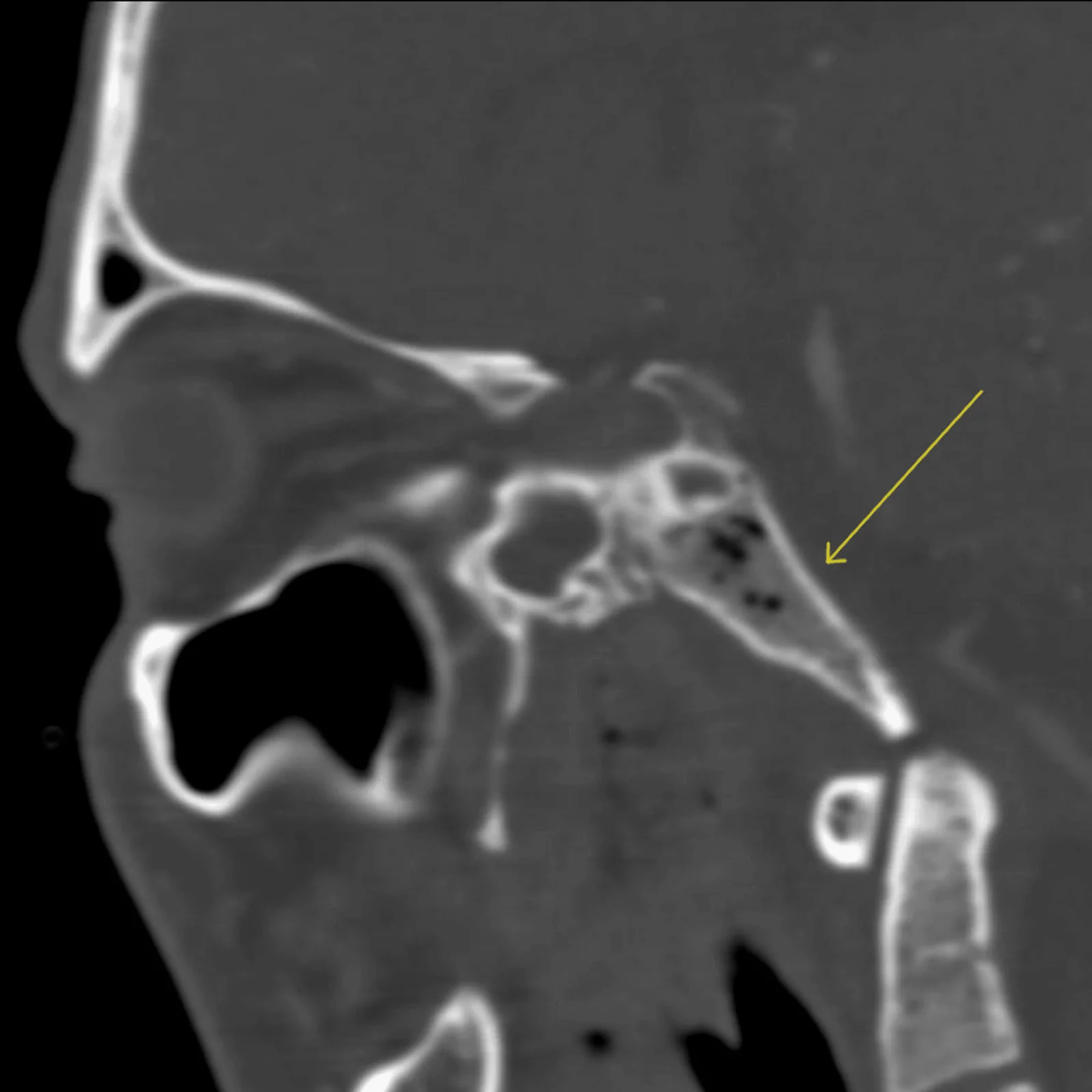
Sagittal CT, with the arrow indicating extensive central bony destruction of the clivus with pockets of intraosseous gas, consistent with osteomyelitis CT, computed tomography

**Figure 6:**
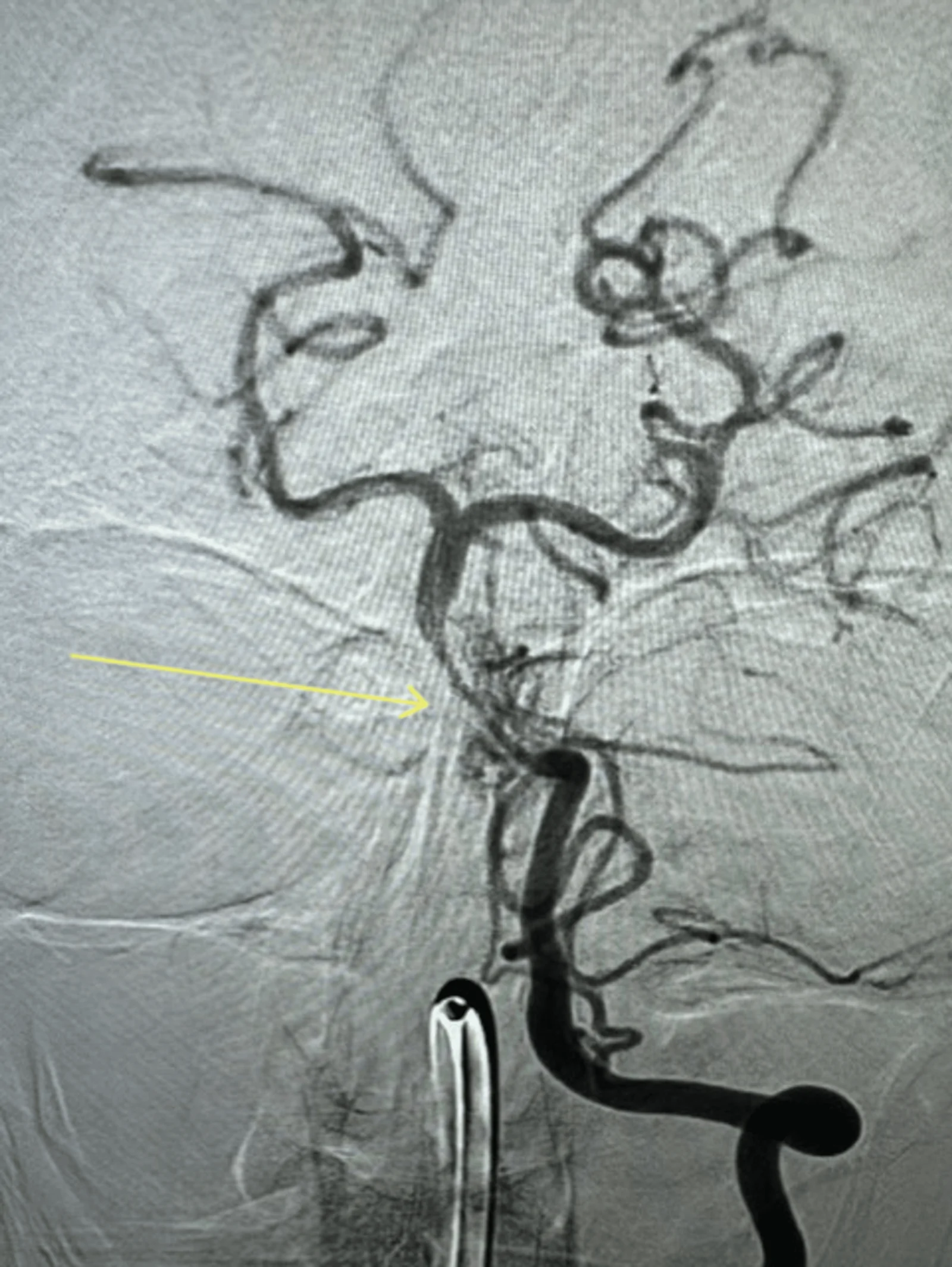
DSA, with the arrow indicating dysmorphic narrowing of the right internal carotid artery. Severe luminal irregularity extends from the high cervical segment to the supraclinoid portion DSA, digital subtraction angiography

Repeat chest computed tomography at the tertiary center showed bilateral nodular consolidations, some with internal cavitation. In the clinical context, these pulmonary lesions were considered suggestive of septic emboli. An extensive infectious evaluation, including blood and cerebrospinal fluid cultures and fungal and atypical infectious testing, was initially unrevealing. Microbial cell-free DNA sequencing of blood subsequently identified *F. necrophorum*, which was also detected by molecular testing of cerebrospinal fluid and an operative sphenoid sinus specimen. Imaging demonstrated clival osteomyelitis with an epidural abscess, and surgical exploration revealed mucopurulent discharge in the sphenoid sinuses. Together with right internal jugular vein thrombosis, these findings supported a diagnosis of atypical Lemierre’s syndrome despite the absence of preceding pharyngitis.

The patient’s presentation with thrombocytopenia, multifocal infarctions, and widespread thrombosis prompted consideration of several alternative diagnoses. Initially, immune thrombocytopenia was suspected at the outside hospital, and he was treated with corticosteroids. In retrospect, the concurrent systemic illness, leukocytosis, acute kidney injury, and later confirmation of disseminated *F. necrophorum* infection favored infection-associated thrombocytopenia, with a possible consumptive component. Disseminated intravascular coagulation (DIC) and thrombotic thrombocytopenic purpura (TTP) were considered in the differential diagnosis but were not established, as thrombocytopenia and thrombosis alone do not establish DIC. Furthermore, the combination of arterial and venous thromboses prompted evaluation for a hypercoagulable or autoimmune process, including antiphospholipid syndrome and systemic vasculitis. The evaluation did not establish an alternative noninfectious diagnosis.

Transesophageal echocardiography demonstrated a bicuspid aortic valve with mild regurgitation and an echodensity at the fused-cusp raphe, favored to represent leaflet thickening or calcification. No independently mobile vegetation or paravalvular abscess was identified, although an atypical vegetation could not be excluded. Bubble study showed mild transpulmonary shunting. These findings did not establish infective endocarditis.

Invasive fungal infection was also considered because of the pulmonary nodules and corticosteroid exposure. Lung biopsy showed inflammation and necroinflammatory debris without evidence of vasculitis; fungal and acid-fast bacilli stains were negative. Progressive disease despite broad antimicrobial therapy did not exclude Fusobacterium infection, particularly in the setting of an unresolved infectious focus and thrombotic complications.

While the diagnostic evaluation was ongoing, the patient received broad-spectrum antibacterial, antifungal, and antiviral therapy for suspected disseminated infection. Following identification of *F. necrophorum*, antimicrobial therapy was transitioned to meropenem monotherapy and subsequently narrowed to ceftriaxone and metronidazole for a planned prolonged course. Because cultures remained negative, susceptibility testing was not available; antibiotic selection was empirical and based on expected *F. necrophorum* susceptibility patterns and the need for central nervous system and skull-base penetration.

The patient underwent endoscopic surgical debridement of the sphenoid sinus and clivus for source control. Given the extent of venous and arterial thrombosis and evidence of ongoing embolic phenomena, therapeutic anticoagulation was initiated with heparin and later transitioned to low-molecular-weight heparin.

Supportive care included multidisciplinary management involving neurology, infectious disease, otolaryngology, and rehabilitation services, including speech and swallow therapy and physical therapy.

Following targeted antimicrobial therapy and surgical source control, the patient demonstrated gradual clinical improvement, with resolution of fevers and normalization of inflammatory markers. Neurological deficits stabilized but did not fully resolve, with persistent left-sided weakness, dysphagia, and right Horner’s syndrome at the time of discharge.

The patient was discharged to an acute rehabilitation facility for ongoing therapy, with a prolonged antimicrobial course consisting of intravenous ceftriaxone and oral metronidazole, as well as continued anticoagulation.

## Discussion

Lemierre’s syndrome is a rare but severe infection most commonly caused by *F. necrophorum*, classically characterized by septic thrombophlebitis of the internal jugular vein with subsequent septic embolization. Although typically associated with antecedent oropharyngeal infection, atypical presentations involving multiple organ systems may occur and contribute to diagnostic delay [3].

This case highlights several important atypical features. In retrospect, infection-associated thrombocytopenia was favored, with a possible consumptive component; the available data did not establish primary immune thrombocytopenia, DIC, or TTP. Reported mechanisms of thrombocytopenia in Lemierre’s syndrome include Fusobacterium-associated hemagglutinin-mediated platelet aggregation and severe systemic inflammation, and prior reports describe both profound thrombocytopenia and TTP-like presentations [4,5]. Corticosteroids were administered before recognition of the infection. This may have contributed to progression of the underlying infection by suppressing the host immune response, illustrating a critical diagnostic pitfall in patients presenting with thrombocytopenia and systemic illness. However, the contribution of corticosteroid exposure cannot be established from this case.

The predominant neurological manifestations, including arterial occlusion, venous sinus thrombosis, and multifocal ischemic stroke, are uncommon in Lemierre’s syndrome and reflect extensive vascular involvement. Notably, this case demonstrates simultaneous and widespread arterial and venous disease, including internal carotid artery occlusion and cerebral venous sinus thrombosis. While venous thrombophlebitis is a hallmark of Lemierre’s syndrome, significant arterial involvement is less frequently reported and may reflect severe infectious vasculopathy or contiguous spread [6].

The coexistence of *F. necrophorum* infection, internal jugular vein thrombosis, pulmonary lesions suggestive of septic emboli, and extensive skull-base and neurovascular involvement is consistent with both hematogenous dissemination and contiguous spread. The sequence of these events remains uncertain.

The presence of clival osteomyelitis represents an uncommon manifestation and suggests contiguous extension of infection to the skull base. Given the proximity of the clivus to critical neurovascular structures, this may have contributed to both cranial neuropathies and arterial complications observed in this case.

The lymphocytic predominance in cerebrospinal fluid further complicated early recognition and broadened the differential diagnosis to include viral, fungal, and autoimmune etiologies. Similarly, pulmonary nodules and immunosuppression raised concern for invasive fungal infection, and negative lung biopsy findings contributed to diagnostic uncertainty.

The diagnosis was supported by molecular detection of *F. necrophorum* alongside the clinical and imaging findings, highlighting the utility of advanced molecular diagnostics in cases of disseminated infection with inconclusive conventional testing.

Taken together, this case illustrates a severe and atypical form of Lemierre’s syndrome characterized by diagnostic delay, immunosuppression, and widespread vascular involvement. The combination of corticosteroid exposure, skull base infection, and simultaneous arterial and venous thromboses suggests a multifactorial pathophysiology involving both hematogenous dissemination and contiguous spread, resulting in extensive neurovascular injury.

Similar cases in the literature have described rare neurological complications of Lemierre’s syndrome, including cavernous sinus thrombosis, arterial involvement, and skull base osteomyelitis. Clival osteomyelitis has been reported in only a limited number of cases [7,8]. Compared with prior reports, this case is notable for the combination of extensive arterial and venous thromboses, an atypical initial presentation mimicking immune thrombocytopenia, and delayed diagnosis despite broad evaluation. These features highlight the potential for Lemierre’s syndrome to present with predominant neurovascular complications and mimic noninfectious conditions, underscoring the importance of maintaining clinical suspicion in patients with unexplained thrombosis and systemic illness.

## Conclusions

This case illustrates that Lemierre’s syndrome can present with extensive arterial and venous neurovascular complications despite the absence of classic oropharyngeal symptoms. In a young patient with systemic illness, thrombocytopenia, and evolving neurological deficits, occult infection should remain in the differential diagnosis rather than attributing the presentation solely to a primary hematologic or autoimmune disorder. Skull-base infection accompanied by multifocal infarction or venous thrombosis should prompt evaluation for contiguous vascular involvement.

In this patient, bacterial infection was identified despite lymphocyte-predominant cerebrospinal fluid and negative conventional cultures, with molecular testing helping to establish the diagnosis. The case underscores the importance of reassessing an initially noninfectious diagnosis as the clinical course evolves and coordinating antimicrobial treatment and source control across specialties. Persistent neurological deficits despite treatment highlight the potential for lasting consequences of this presentation.
